# Comparison of long and short 16S rRNA gene amplicon sequencing for agricultural soil and grain microbiomes using custom Kinnex libraries

**DOI:** 10.1093/ismeco/ycag282

**Published:** 2026-10-06

**Authors:** Sung-Jong Lee, Tiffany Chin, Niradha Withana Gamage, Janice Bamforth, Luke Dojack, Salvador Osuna-Caballero, Curtis Pozniak, Kirstin E Bett, Sean Walkowiak

**Affiliations:** Grain Research Laboratory, Canadian Grain Commission, Winnipeg, MB, R3T 6C5, Canada; Grain Research Laboratory, Canadian Grain Commission, Winnipeg, MB, R3T 6C5, Canada; Grain Research Laboratory, Canadian Grain Commission, Winnipeg, MB, R3T 6C5, Canada; Grain Research Laboratory, Canadian Grain Commission, Winnipeg, MB, R3T 6C5, Canada; Crop Development Centre, University of Saskatchewan, Saskatoon, SK, S7N 5A8, Canada; Crop Development Centre, University of Saskatchewan, Saskatoon, SK, S7N 5A8, Canada; Crop Development Centre, University of Saskatchewan, Saskatoon, SK, S7N 5A8, Canada; Crop Development Centre, University of Saskatchewan, Saskatoon, SK, S7N 5A8, Canada; Grain Research Laboratory, Canadian Grain Commission, Winnipeg, MB, R3T 6C5, Canada

**Keywords:** microbiome, soil, grain, long read, PacBio, Kinnex

## Abstract

Bacteria are an important component of environmental microbiomes, contributing to animal, plant, and soil health. The 16S rRNA gene is used as a barcode for taxonomic classification of bacteria and analysis of bacterial diversity in environmental samples. The 16S barcode region is ~1500 bp and has nine hypervariable regions (V1-V9) that differ in their ability to distinguish various taxa. Short-read sequencing approaches enabled the targeting of subsets of these variable regions; however, long-read sequencing platforms can span all nine hypervariable regions, resulting in greater taxonomic resolution. We explored different DNA extraction procedures, DNA polymerases, and amplicon lengths to determine their effects on taxonomic resolution. We compared the full-length 16S barcode region (V1-V9) and subsets of the barcode (V3-V5 and V4-V5) using custom amplicons in the Pacific Biosciences (PacBio) Kinnex long-read sequencing platform, which has enhanced output over traditional PacBio amplicon sequencing. To capture differences in DNA quality and microbial complexity, two types of environmental DNA samples were assessed: 32 samples from agricultural soil and another 32 samples cultured from grain. The study demonstrates that custom amplicon sequencing using the PacBio Kinnex system can be implemented with relative ease, allowing for increased outputs over traditional custom amplicon sequencing. The greatest taxonomic resolution was achieved with longer amplicons; however, interactions between DNA quality, amplicon lengths, chimeric reads, community complexity, and taxonomic assignment/resolution should be considered. The samples themselves also represent unique microbiomes that are not well represented in the literature, adding to our knowledge of diverse microbes on grain and in soils.

## Introduction

Amplicon sequencing of the 16S rRNA gene from bacterial communities in agricultural samples, such as bulk soil, rhizosphere soil, plant roots, water and food products, etc., enables comparisons to databases of reference bacterial 16S rRNA sequences and the identification of bacteria present in complex samples [1–3]. Count information for each barcode can then be used to determine the quantity of each community member and diversity of the bacterial communities for each sample [1–3]. Studies of soil microbiomes have uncovered massive amounts of diversity; as an example, one study identified over 79 000 bacterial operational taxonomic units (OTUs) from soil across 24 European countries [4].

The 16S rRNA gene is ~1500 bp in length and has nine hypervariable regions (V1-V9) that are used as targets for bacterial taxonomic identification. Amplicon sequencing of the 16S rRNA gene became well established for taxonomic analysis in microbiome studies for a wide array of sample types but was limited to focusing on one or two hypervariable regions in each study due to sequencing read-length limitations [5]. For example, the V4 region (515F/806R) was selected for global patterns of 16S rRNA diversity across diverse clinical and environmental samples [2]. Soil microbiome analyses were also conducted using the region of V3-V4 (341F/785R) to investigate the peanut rhizosphere [3], and the V4-V5 (515F/926R) region was used to examine diverse topsoil microbiomes [1]. While these studies provide valuable information on microbiome communities, targeting only a subset of the variable regions has limitations, reducing taxonomic resolution and making it more difficult to compare results between studies that use different combinations of hypervariable regions.

With the advent of accurate high-fidelity (HiFi) long-read sequencing with Pacific Biosciences (PacBio) sequencing, it is now possible to target all nine hypervariable regions in a single amplicon, thereby increasing taxonomic resolution and universality of the amplicon sequencing approach [6]. Several recent studies have compared short and full-length 16S amplicons from bacterial custom mock communities and some human samples such as saliva, oral biofilm, and feces [7–10]. Long-reads assess more hypervariable regions than shorter reads and therefore have improved taxonomic resolution. However, studies of clinical samples indicate that there may still be some practical advantages to targeting a subset of regions, such as instrument availability and costs, particularly in cases where the additional variable regions captured by long-reads do not add additional discriminatory power for certain taxa of interest [5, 6]. While the subset of variable regions that are most informative for human clinical studies may be different than those for agricultural samples including soils, longer amplicons that capture more hypervariable regions have also been demonstrated to improve taxonomic resolution in agricultural samples [9]. Veselovsky *et al.* (2025) compared the amplicons of V4 and V4-V5 regions to the same regions *in silico* trimmed from the full-length amplicon (V1-V9) from soil DNA samples and demonstrated that if the same hypervariable regions were partially selected for amplification, the taxonomic outputs would be similar between the amplicons and *in silico* amplicons. Altogether, these studies suggest that sequencing the full-length 16S amplicon has greater taxonomic resolution than short-read amplicon sequencing (such as V4, V3-V4, V4-V5 or V3-V5), is reproducible, and it may be possible to perform comparisons of long-read sequencing data to previous datasets that used subsets of these hypervariable regions.

PacBio HiFi sequencing can generate reads with lengths of ~20 000 bp and an accuracy of 99.9%, which is a marked improvement over early generations of long-read sequencing [8]. Standard amplicon sequencing on a PacBio instrument of an amplicon of only ~1500 bp would, therefore, be inefficient and lead to suboptimal sequencing yields (Fig. 1A). Recent Kinnex library preparation protocols employ modifications to the standard library preparation to allow for amplicons to be concatenated into longer molecules that can be sequenced as a single read on the PacBio instrument, thereby increasing yields (Fig. 1B). The PacBio Kinnex library preparation protocol has been optimized with kits available specifically for 16S amplicon sequencing (PacBio). By tethering multiple amplicons together, sequencing output has increased significantly and has been successfully applied to different sample types, including insect gut and soil microbiota [11, 12].

**Figure 1:**
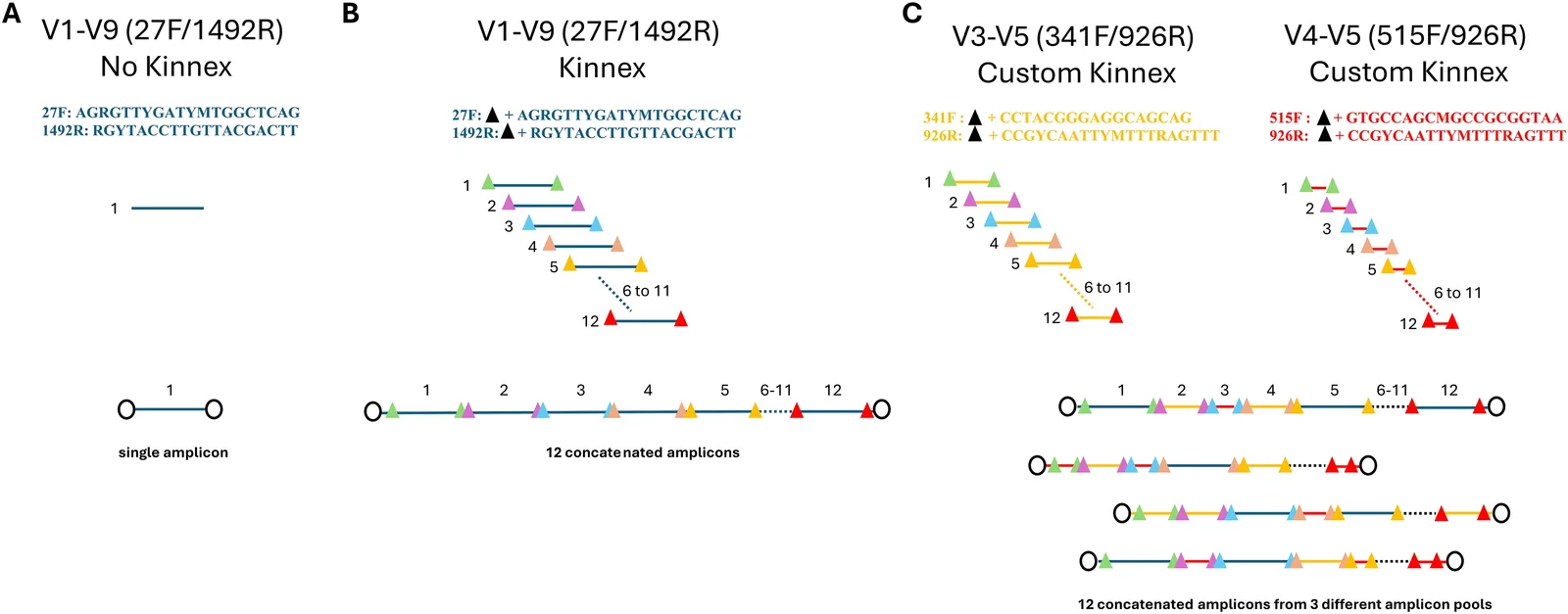
Amplicon sequencing of the V1-V9 (27F/1492R, blue) region as a single amplicon (A) and as a Kinnex library that concatenates 12 amplicons together (B); colored triangles represent breakpoints between different amplicons that have been concatenated together and the black triangles represent the standard Kinnex barcodes. (C) Customization of the Kinnex protocol to different amplicons for V3-V5 (341F/926R, purple) and V4-V5 (515F/926R, red) and combining them together into a single library that concatenates mixed amplicons.

While longer amplicons have higher taxonomic resolution, amplification of longer fragments can lead to other challenges. There are many factors that may bias the results of amplicon sequencing studies, including differences in DNA extraction protocols, primer affinity, PCR parameters, and GC content [13, 14]. One challenge with long-read sequencing is that DNA extraction from some substrates, such as soil, can be difficult and lead to DNA degradation, which could affect downstream processes such as amplification and sequencing of longer DNA sequences [15]. Further, amplification of longer fragments may be prone to chimeric amplicons, particularly if DNA is too fragmented or degraded, sequences are similar, and the number of PCR cycles are increased [16, 17]. Chimeric amplicons are artificial sequences created from two or more templates combining during PCR amplification, which can lead to errors and misidentifications. Some of these biases and challenges may be unavoidable, while others can be tested and controlled. Technologies and protocols for DNA extraction and amplification of the 16S rRNA gene for taxonomic identification continue to improve and diversify, but are often under-described in scientific reports, making it difficult to interpret and compare studies and their biases.

In this study, we focused on investigating the relationships between DNA extraction approaches, DNA quality, amplicon lengths, and taxonomic resolution for soil microbiomes and microbiomes cultured from grains. We customized the PacBio Kinnex library preparation method for the concatenation of amplicons that target different hypervariable regions of the 16S barcode sequence. We also report on our methods in detail and investigate approaches to understand potential sources of sequencing and analysis biases to foster a deeper understanding into best practices for amplicon sequencing.

## Materials and methods

### DNA extraction from soil, wheat grain, and mock microbiome samples

Thirty-two independent soil samples were collected from farm fields near Saskatoon, Saskatchewan, Canada, at pre-seeding in May 2025. The farmland was previously sown with different lentil breeding material and harvested in 2024. Each soil sample was collected at a depth of 15 cm after removing loosely covered topsoil (~2 cm) using a soil probe (tubular) with a 2.5 cm diameter following the Lee *et al.* (2026) protocol. Briefly, three soil samples were collected and combined for each 1.4 × 3.6 m plot and placed into a plastic bag and stored in a cooler with ice packs. The collected soil sample was homogenized into small particles in a plastic bag with a mallet, sub-sampled into a 2 ml microcentrifuge tube, and stored at −20°C [18]. The soil DNA was extracted from the soil sample using the DNeasy PowerSoil Pro Kit (Qiagen) according to the manufacturer protocol, with minor modifications. 0.25 g of soil was added to the PowerBead Tube (Qiagen), followed by the addition of the lysis solution. Samples were processed using a mixer mill (Retsch) with a 20 Hz frequency for 10 minutes. The speed and time in a mixer mill were chosen by considering soil DNA quality and yield based on agarose gels and Qubit dsDNA assay after testing a range of milling conditions (Thermo Fisher Sci.) (Supplemental Fig. 1 and Supplemental Fig. 2). We also observed differences in the levels of certain taxa at lower milling frequencies, including reduced levels of Gram-positive bacteria, which stabilized after 10 Hz was used (results not shown).

For the microbial community analysis of wheat grains, 32 independent liquid cultures were generated using Health Canada’s Compendium of the Laboratory Procedures for the Microbiological Analysis of Foods (MFLP-52, 2014), with slight modifications [19]. The wheat grain samples were obtained from the Harvest Sample Program (HSP) of the Canadian Grain Commission (CGC). Briefly, 25 g of grain was transferred into a 400 ml sterile blender bag with a filter (Innovation Diagnostics) and 50 ml buffered peptone water was added to each blender bag. The grain samples were then incubated for 24 h at 37°C, and 175 ml of tryptone soya broth (TSB) was then added to the blender bag. The blender bag was homogenized using a pulsifier (Microgen Bioproduct) for 2 minutes and incubated at 42°C overnight. Given the microbiomes enriched from grain were in liquid cultures, homogenization of the sample using a mixer mill was not necessary. The grain microbiome DNA was extracted from the aliquot of the culture liquid using the NucleoSpin® 96 Tissue kit as per the manufacturer’s instructions (Takara Bio USA, Inc.).

A bacterial mock community was also tested, ZymoBIOMICS Microbial Community DNA Standards (Zymo Research), which was used to evaluate taxonomic performances depending on the variable regions and PCR Mastermixes.

### PCR amplification and Kinnex barcoded library preparation

The primer sets and PCR condition of the 16S amplicons was the same as used in soil and grain microbiome DNA. All of the PCR primers contained the PacBio Kinnex adaptors and barcodes from 16S rRNA amplicon protocol, according to PacBio specifications (“Kinnex libraries from 16S rRNA amplicons”, PacBio). The PCR products were amplified using the three Kinnex barcoded primer pairs, which had the fixed sequences 16S 27F/1492R (27F: AGRGTTYGATYMTGGCTCAG/1492R: RGYTACCTTGTTACGACTT), 341F/926R (341F: CCTACGGGAGGCAGCAG/926R: CCGYCAATTYMTTTRAGTTT) and 515F/926R (515F: GTGCCAGCMGCCGCGGTAA) (Fig. 1; Supplemental Table 1). Amplification was performed using one of three different PCR master mixes, including the KAPA HiFi HotStart ReadyMix (KAPA, Roche), Phusion HotStart II HiFi PCR MasterMix (Phusion, Thermo Fisher Sci.) and Platinum HotStart PCR MasterMix (Platinum, Thermo Fisher Sci.). The PCR conditions followed the standard protocol for preparing Kinnex libraries from 16S rRNA amplicons (PacBio), which was performed with 20 cycles and an annealing temperature of 57°C, as recommended, after assessing different cycling conditions (Supplemental Fig. 3). The 20 μl of PCR reaction contained 1.6 ng DNA template, 0.3 μM forward and reverse primers each and 10 μl of a 2× PCR master mix. The PCR was performed in the 384-well thermocycler (Veriti, Thermo Fisher Sci).

After pooling equal volumes of the PCR products amplified by the three primer sets and the three PCR master mixes, the pooled PCR products were cleaned by SMRTbell Cleanup beads (PacBio). The cleaned barcoded PCR products were used to prepare libraries using the Kinnex concatenation kit (PacBio) and Kinnex 12-fold kit (PacBio). Each individual PCR reaction was distinguished by 288 barcode combinations. Each DNA sample was amplified by the three primer pairs and the three PCR master mixes, respectively (3 primer pairs ×3 master mixes ×32 DNA samples = 288 Kinnex barcode combinations). Adapters were added to the soil and grain microbiome DNA samples using the Kinnex adaptors bc01 and bc02 (PacBio), respectively, and sequenced in the same flowcell (SMRT Cell 8 M, PacBio). All samples were combined with equimolar (based on length) amounts as a single library for sequencing. In total, the 576 combinations of the barcodes and the adaptors were sequenced on a single flowcell using a Sequel IIe sequencer (PacBio).

### 16S amplicon sequencing data processing

The SMRT Link v13.0 (PacBio) and PacBio HiFi 16S Nextflow pipeline (https://github.com/PacificBiosciences/HiFi-16S-workflow) was used to demultiplex and analyze the Kinnex sequencing data. Kinnex reads were separated by barcodes using the Read Segmentation data utility tool from the SMRT Link v13.0 (PacBio). The barcoded reads were demultiplexed using the Demultiplex Barcodes Data utility tool from the SMRT Link v13.0 (PacBio). Sequencing data quality were assessed using seqkit with a quality value cut-off of Q20 [20]. Primers were removed using cutadapt [21]. In the case of *in silico* V3-V5 and V4-V5 regions, primers from 341F/926R and 515F/926R were used in V1-V9 to remove primers using cutadapt. Samples were compared with and without primer removal. Sequencing data were imported into the QIIME 22022.2 environment [22], and denoised using DADA2 into amplicon sequence variants (ASVs) [23]. Samples and reads were filtered and trimmed using DADA2 with a minimum quality of 1 and a maximum expected error of 2. Chimera detection was done using removeBimeraDenovo function from DADA2. Taxonomic classification of ASVs was done using both a VSEARCH and a Naïve-Bayes (NB) classifier method. In the VSEARCH method for 16S data, the Silva 16S database with a percent identity of 97% was used [24]. For the NB classifier method, Silva, GTDB version 220, and GreenGenes2 version 2024.09 reference databases were used, with the priority order of assignment being GreenGenes2, GTDB, and Silva [24–26].

## Results and discussion

Sequencing the full-length 16S gene with PacBio HiFi and Kinnex technologies has benefits of high accuracy and data outputs, but few studies have applied Kinnex to custom amplicons [8]. Here, we describe the sequencing of custom 16S amplicons using these tools in order to provide precedence that they can be applied to any sequences or genes (Fig. 1). While previous amplicon sequence approaches targeted a single copy of a single amplicon (Fig. 1A), the Kinnex protocol concatenates 12 amplicons together, thereby increasing sequencing output (Fig. 1B). We have adapted this method to custom amplicons (Fig. 1C), which allows for custom amplicons to be concatenated and sequenced with enhanced outputs. Further, we combined three separate amplicons together, which allowed for different amplicons to be concatenated to one another during library preparation (Fig. 1C). An advantage of combining amplicons is that this can allow for enhanced multiplexing, where amplicons for different target regions can be combined and segregated post-processing. Specifically in this study, we focus on the 16S gene and investigate three different amplicons of different sizes, covering different variable regions. We also explore these different amplicons in the context of complex interactions between DNA quality and sample complexity, as well as explore methods for DNA extraction, target amplification, and data processing/analysis.

### Adjusting DNA extraction, DNA polymerase, and cycling conditions affects 16S amplification

Methods for DNA extraction have been demonstrated to impact DNA quality and amplicon sequencing results, with amplification of chimeric sequences in degraded environmental DNA samples being of particular concern for similar targets and longer amplicons, with chimeras potentially occurring at levels >45% [15, 16, 27]. Previous studies have reported varying performance of soil DNA extraction kits and methods in obtaining high-quality DNA, with the Qiagen Power Soil Pro Kit being well-suited to high molecular weight DNA extraction [15], which was used in this study.

To better understand and minimize potential biases leading to DNA shearing and degradation, we performed an initial assessment of the effects of adjusting frequency and length of the mixing during homogenization on DNA quality and yields (Supplemental Fig. 1 and Supplemental Fig. 2). As expected, there was a positive relationship between DNA fragmentation and overall yield when increasing milling frequency and time. We selected milling conditions of 20 Hz and 10 minutes that were optimal for yield while maintaining sufficient large DNA fragments for amplicon sequencing. Quality analysis indicated that the soil DNA had a size range distribution from 4000 to 15 000 bp with a peak at 8000 bp, while the DNA from the microbial communities enriched from grains ranged from 7000 to 45 000 bp and had a peak of about 17 000 bp (Supplemental Fig. 2).

Prior to preparing the amplicon sequencing libraries, the soil and grain microbiome DNA samples were confirmed to have the expected amplicon lengths (Supplemental Fig. 3). In addition, the three different amplicons were successfully amplified by the three DNA polymerases (KAPA, Phusion and Platinum). The latest protocols for 16S amplicon sequencing by PacBio recommended the use of the KAPA polymerase, but when investigating other gene targets in soil DNA, the Phusion and Platinum polymerases also performed well in terms of the yield and specificity of PCR products (results not shown). While the Platinum polymerase is not a HiFi DNA polymerase, it is hot-start and was expected to perform well for amplification under challenging PCR conditions, such as DNA from soil. Oxford Nanopore Technologies sequencing and earlier generations of PacBio sequencing have lower read accuracies than short-read sequencing technologies; nevertheless, pipelines have been developed that can accommodate reads with lower accuracy that may be accommodating of a lower fidelity polymerase [28].

For all three polymerases, between 20 and 23 PCR cycles resulted in adequate amounts of amplification for sequencing. For the Kinnex library preparation, to minimize PCR artifacts and chimeras, we opted to perform 20 PCR cycles, which is the recommended number of cycles in the protocols from PacBio. We note that there were some qualitative differences between the DNA polymerases and master mixes, with the Phusion DNA polymerase resulting in greater amplification and the Platinum resulting in more refined product sizes (Supplemental Fig. 3). After amplicon sequencing, we observed differences in the total outputs depending on which of the three DNA polymerases and master mixes were used (Table 1). The greatest sequencing outputs (total sequencing reads) were from the Phusion polymerase, followed by the KAPA polymerase, and then the Platinum polymerase (Fig. 1). These findings match the qualitative observations of the PCR products from the DNA polymerases and may reflect differences in PCR efficiencies and outputs (Supplemental Fig. 3). While the trends were consistent across all amplicon sizes, the magnitude of the effect did appear to change across amplicons and sample types, possibly due to differences in the primers and sequences of the targets as well as the quality of the DNA between sample types. We note that the KAPA polymerase, despite being the polymerase recommended for 16S amplicon sequencing, had lower output than the Phusion polymerase, particularly for 27F/1492R (Fig. 1).

**Table 1:** Taxonomic assignments for the ZymoBIOMICS microbial community DNA standard (mock community).

	**Expected composition** ^**a**^	**27F/1492R**			**341F/926R**			**341F/926R *in silico*** ^**c**^			**515F/926R**			**515F/926R *in silico*** ^**c**^		
**Species**		**KAPA**	**Phusion**	**Platinum**	**KAPA**	**Phusion**	**Platinum**	**KAPA**	**Phusion**	**Platinum**	**KAPA**	**Phusion**	**Platinum**	**KAPA**	**Phusion**	**Platinum**
*Pseudomonas aeruginosa*	4%	3%	3%	2%	3%	3%	3%	3%	3%	2%	3%	3%	3%	3%	3%	2%
*Escherichia coli*	10%	8%	9%	7%	8%	8%	7%	8%	9%	7%	8%	8%	7%	8%	9%	7%
*Salmonella enterica*	10%	8%	10%	8%	8%	8%	8%	8%	9%	8%	8%	8%	7%	8%	9%	8%
*Lactobacillus fementum* ^b^	18%	15%	16%	16%	18%	18%	18%	15%	17%	17%	19%	20%	18%	15%	17%	16%
*Enterococcus faecalis*	10%	11%	10%	12%	12%	11%	12%	11%	10%	12%	11%	11%	12%	12%	11%	12%
*Staphylococcus aureus*	16%	19%	17%	19%	16%	16%	14%	18%	17%	19%	16%	16%	15%	19%	18%	19%
*Listeria monocytogenes* ^d^	14%	13%	15%	17%	17%	16%	17%	16%	15%	17%	17%	15%	17%	16%	15%	16%
*Bacillus subtilis* ^b^ ^,^ ^e^	17%	20%	18%	19%	20%	21%	21%	20%	19%	20%	19%	20%	21%	19%	19%	20%

^a^Expected composition is based on the manufacturer description of the mock community content.

^b^New taxonomic names in the NCBI datasets (NCBI reference accession No. CP118021/CP118022 & CP132481) for *Lactobacillus fementum* and *B. subtilis* are *Limosilactobacillus fementum* and *Bacillus spizizenii*, respectively.

^c^ *In silico* amplicons obtained from the 27F/1492R sequences.

^d^Amplicons were assigned as *Listeria seeligeri* for 515F/926R, which is consistent with expected assignment based on the variable region used.

^e^Amplicons were assigned as *B. halotolerans* or *B. spizizenii* for 341F/926R and as *Bacillus halotolerans* for 515F/926R, all are consistent with expected assignments based on the variable regions used.

### Capturing additional variable regions improves taxonomic classification

Amplification of some target PCR amplicons, such as the ~1500 bp full-length 16S rRNA barcode from environmental DNA, can be challenging due to the possibility of fragmented DNA, the complexity of microbial communities, and high sequence similarities across species [13, 14, 16]. Here, we explored the use of three different amplicon lengths on taxonomic classification for two different sample types, soils and cultured microbial communities from grain, that have differences in DNA quality and community composition. Using different DNA polymerases did not appear to affect reads passing filters or occurrence of chimeras (Supplemental Table 2). There were differences in the number of ASVs between polymerases, but this was likely a result of differences in input reads and not polymerase activity. After normalizing for number of reads, the Platinum polymerase may have had slightly elevated ASVs in some cases, perhaps due to its lower fidelity (Supplemental Table 2). The results for the mock community were also consistent for all three polymerases and were similar to the expected ratios for the species present (Table 1). Since the polymerase used appeared to have minimal effects on the taxa identified, amplicon sequencing data were pooled across the polymerases and the data from each primer pair were processed for taxonomic assignment (Table 2).

**Table 2:** Sequencing outputs and taxonomic assignments for different amplicons from soil and microbial communities cultured from grain.

**Sample type**	**Statistic**	**27F/1492R**			**341F/926R**	**515F/926R**
		**(V1-V9)**	**(V3-V5 *in silico*)** ^**a**^	**(V4-V5 *in silico*)** ^**a**^	**(V3-V5)**	**(V4-V5)**
Soil	Dataset count	96	96	96	96	96
	Total CCS reads	2 196 483	2 196 483	2 196 483	4 190 478	5 846 213
	Total DADA2 input (trimmed)	2 140 598	2 055 838	2 149 349	4 076 940	5 649 121
	Total non-chimeric reads	1 661 625	1 733 136	1 946 628	3 512 354	5 186 111
	Total ASVs found	30 036	17 863	14 504	25 462	22 919
	**(Classification using VSEARCH)**					
	ASVs classified at species level	9844	6921	4976	9192	7053
	% reads in ASVs at species level	47%	59%	55%	58%	54%
	Mean assignment confidence at Species level (range)	98.9% (51.8%–100%)	98.3% (51.1%–100%)	97.4% (51.9%–100%)	98.3% (51.1%–100%)	97.4% (51.9%–100%)
	ASVs classified at genus level	10 208	7627	5775	10 106	8192
	% reads in ASVs at genus level	49%	72%	72%	70%	70%
	Mean assignment confidence at genus level (range)	98.9% (51.8%–100%)	98.2% (51.1%–100%)	97.1% (51.9%–100%)	98.2% (51.1%–100%)	97.1% (51.9%–100%)
	**(Classification using NB)**					
	ASVs classified at species level	16 732	7975	5412	10 993	7945
	% reads in ASVs at species level	54%	44%	39%	44%	38%
	Mean assignment confidence at Species level (range)	96.2% (80%–100%)	95.3% (80%–100%)	94.5% (80%–100%)	95% (80%–100%)	94.5% (80%–100%)
	ASVs classified at genus level	23 833	12 154	8772	16 843	13 108
	% reads in ASVs at genus level	85%	77%	60%	75%	69%
	Mean assignment confidence at Genus level (range)	94.4% (7%–100%)	92.5% (100%)	91.3% (4%–100%)	92.4% (3%–100%)	91.2% (3%–100%)
Grain	Dataset count	95^b^	95^b^	95^b^	96	96
	Total CCS reads	2 407 057	2 407 057	2 407 057	7 062 229	8 855 093
	Total DADA2 input (trimmed)	2 350 753	2 378 678	2 371 863	6 918 792	8 651 626
	Total non-chimeric reads	1 741 886	2 123 728	2 221 312	6 256 285	8 114 346
	Total ASVs found	2606	603	370	869	595
	**(Classification using VSEARCH)**					
	ASVs classified at species level	2453	492	298	704	474
	% reads in ASVs at species level	98%	82%	88%	82%	62%
	Mean assignment confidence at Species level (range)	97% (51.4%–100%)	92.3% (52%–100%)	89.4% (53.1%–100%)	92.6% (52%–100%)	89.7% (53.1%–100%)
	ASVs classified at genus level	2518	551	354	791	565
	% reads in ASVs at genus level	100%	100%	100%	100%	100%
	Mean assignment confidence at Genus level (range)	96.9% (51.4%–100%)	92.5% (52%–100%)	89.6% (53.1%–100%)	93% (52%–100%)	90% (53.1%–100%)
	**(Classification using NB)**					
	ASVs classified at species level	2322	412	231	608	364
	% reads in ASVs at species level	96%	91%	60%	91%	62%
	Mean assignment confidence at species level (range)	97.3% (80%–100%)	94% (80%–100%)	94.4% (80%–100%)	94% (80%–100%)	94.2% (80%–100%)
	ASVs classified at genus level	2544	554	336	807	542
	% reads in ASVs at genus level	100%	99%	94%	99%	94%
	Mean assignment confidence at genus level (range)	95.2% (18%–100%)	84.6% (8%–100%)	82.9% (4%–100%)	85.1% (10%–100%)	81.4% (7%–100%)

a The shorter amplicon for (V3-V5) and (V4-V5) were extracted *in silico* from the larger amplicon generated from amplification by 27F/1492R.

b Note, one barcode combination failed for the 27F/1492R (V1-V9) amplicon for the grain microbiome samples.

For both the soil and grain microbiome samples, the shorter amplicons produced more sequencing reads under the same PCR conditions (Table 2 and Fig. 2); the differences in read count suggest that the smaller amplicons were more efficiently amplified than larger amplicons. Despite having fewer reads, the total ASVs-count was greatest for the full-length amplicon (Table 2), thus supporting the notion of increased taxonomic resolution of longer amplicons that capture additional variable regions. The total number of ASVs was much higher in the soil samples than the microbial communities cultured from grain, which was expected given the high level of diversity typically found in soil microbiomes [1, 3, 9]. It is likely that culturing steps would encourage the growth of faster-growing microbes that would dominate the communities in the grain microbiome samples; whereas the soil may be expected to have a more diverse microbiome that would include unculturable microbes.

**Figure 2:**
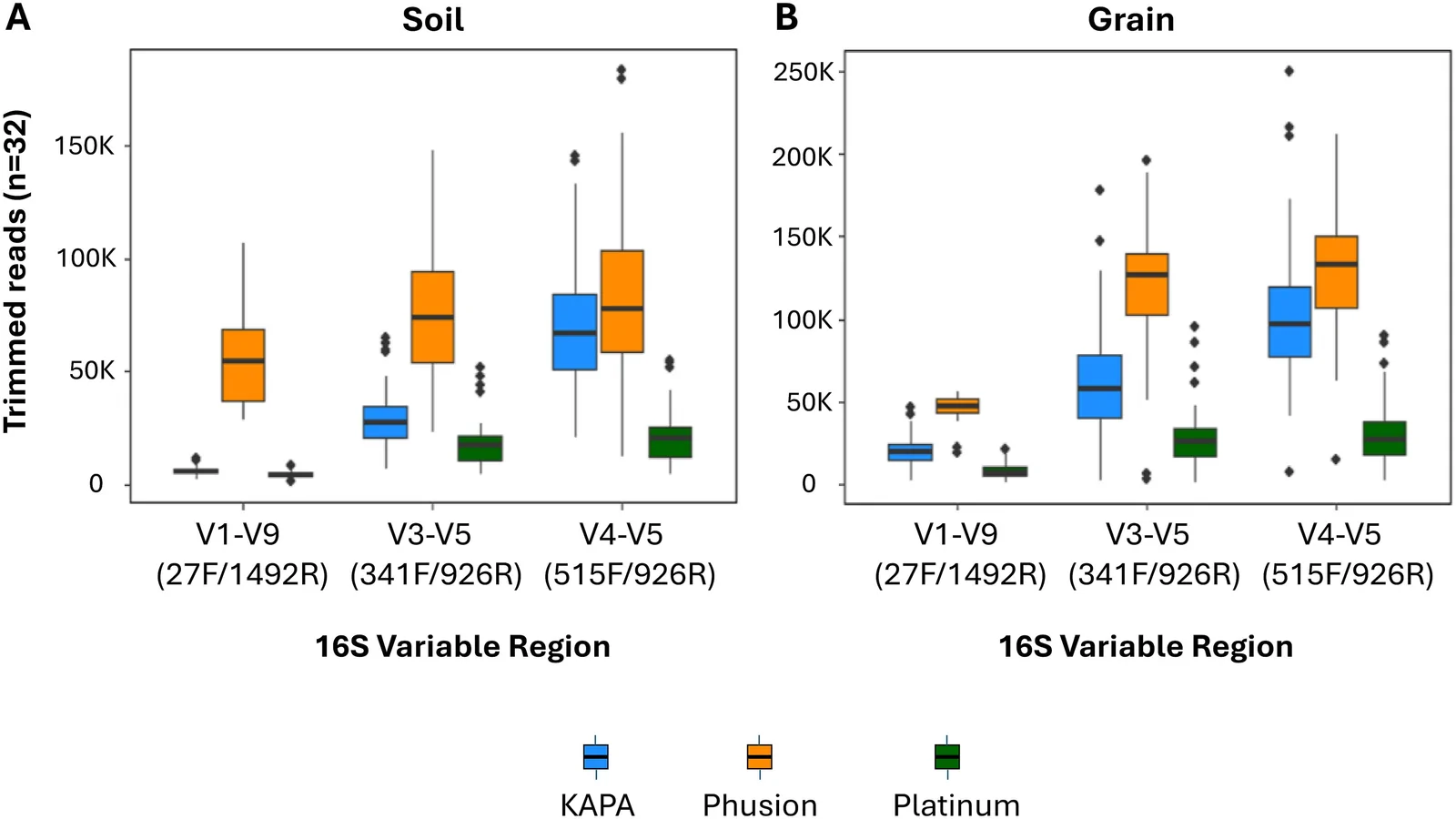
Read counts for PCR products amplified in soil (A) and grain (B) microbiome samples. Three different primers sets (27F/1492R, 341F/926R and 515F/926R) and the three different PCR master mixes (KAPA: KAPA HiFi HotStart ReadyMix (Roche), Phusion: Phusion HotStart II HiFi PCR MasterMix (fisher sci.) and platinum: Platinum HotStart PCR MasterMix (fisher sci.) were used.

Analysis of the ASV classifications at the genus and species level indicates that assignment is amplicon length- and sample-dependent (Table 2). In general, the mean confidence for the two models used for classification was stable across the different amplicons (Table 2). When comparing between technologies at the species and genus level for the soil samples, NB had a lower mean confidence percentage than VSEARCH. For the grain samples, NB had a higher mean confidence percentage at the species level, but a lower mean confidence percentage at the genus level and wider range in confidence. The number of ASVs classified to the species and genus levels for the grain microbiome was greatest in the full-length amplicon, followed by V3-V5 and the V4-V5 (Table 2). The same trend was true for soil, with the exception of the classifications by VSEARCH, though the taxonomic assignments were generally lower in the soil microbiomes compared to the grain microbiomes, likely reflecting increased complexity and incomplete databases for uncultured microbes (Table 2 and Table 3). To further explore the amplicons that could not be assigned, we performed BLAST analyses of the top 50 non-classified ASVs from each amplicon to the NCBI database and the vast majority of the top hits were to uncultured or unassigned microbes (Supplemental Table 3). The greater proportion of species-level assignments for the longer amplicon in the cultured microbiome from grain may be due to the increased resolution of the longer amplicon and the more complete database of barcodes for culturable microbes (Table 3).

**Table 3:** For top 50 ASVs, the percentages of taxonomic assignments by VSEARCH and NB classification to the kingdom, genus and species levels from the different amplicons from the soil and grain microbiome samples.

		**VSEARCH**			**NB**		
**Sample type**	**Amplicon**	**Kingdom**	**Genus**	**Species**	**Kingdom**	**Genus**	**Species**
Soil	27F/1492R (V1-V9)	72%	72%	68%	100%	86%	34%
	27F/1492R (V3-V5) *in silico*^a^	86%	84%	62%	100%	80%	34%
	27F/1492R (V4-V5) *in silico*^a^	96%	86%	70%	100%	72%	40%
	341F/926R (V3-V5)	86%	86%	62%	100%	84%	50%
	515F/926R (V4-V5)	90%	78%	60%	100%	68%	38%
Grain	27F/1492R (V1-V9)	100%	100%	96%	100%	100%	98%
	27F/1492R (V3-V5) *in silico*^a^	100%	100%	90%	100%	98%	80%
	27F/1492R (V4-V5) *in silico*^a^	100%	100%	88%	100%	90%	58%
	341F/926R (V3-V5)	100%	100%	88%	100%	100%	82%
	515F/926R (V4-V5)	90%	100%	88%	100%	92%	60%

a The shorter amplicon for (V3-V5) and (V4-V5) were extracted *in silico* from the larger amplicon generated from amplification by 27F/1492R.

Given that the full-length sequence of 27F/1492R (V1-V9) contained the variable regions captured by the smaller amplicons, we investigated whether we could extract subregions within the amplicon and achieve similar taxonomic results and resolutions to the smaller amplicons targeting those regions independently using different primer pairs (341F/926R and 515F/926R) (Table 2). There were much fewer ASVs identified in the *in silico* subregions for both the samples from soil and grain than the full-length 16S amplicon, supporting the improved resolution of the longer amplicon. Comparisons between the *in silico* extracted subregion and the full-length amplicon also indicated different taxonomic profiles for the soil samples (Fig. 3A). The taxonomic classification of ASVs generally overlapped when comparing the full-length sequence to the *in silico* extracted subregions, albeit at a different taxonomic resolution, with one notable exception. A clear difference was the presence of archaea classifications in the soil samples among the 515F/926R (V4-V5) amplicon sequences, which were 12% using VSEARCH and 16% using NB classifiers. The archaea sequence was not captured by 27F/1492 (V1-V9) or 341F/926R (V3-V5) primers (Fig. 3A), likely due to poor primer binding specificities. The 515F and 806R primers were previously reported and selected to capture both bacterial and archaeal DNA [29, 30], since 515F was paired with 926R in this study, it likely allowed for amplification of authentic targets from archaeal DNA. It is interesting to note, though, that the taxonomic profiles of the less complex samples from the grain microbiomes were less impacted by adjusting the amplicon lengths or by extracting the subregions from the larger amplicon (Fig. 3B), possibly due to lower diversity and fewer archaea. For the mock communities, compositional differences in taxonomic assignment were observed in different amplicon lengths, likely due to ambiguity and overlap between barcodes of related taxa (Table 1).

**Figure 3:**
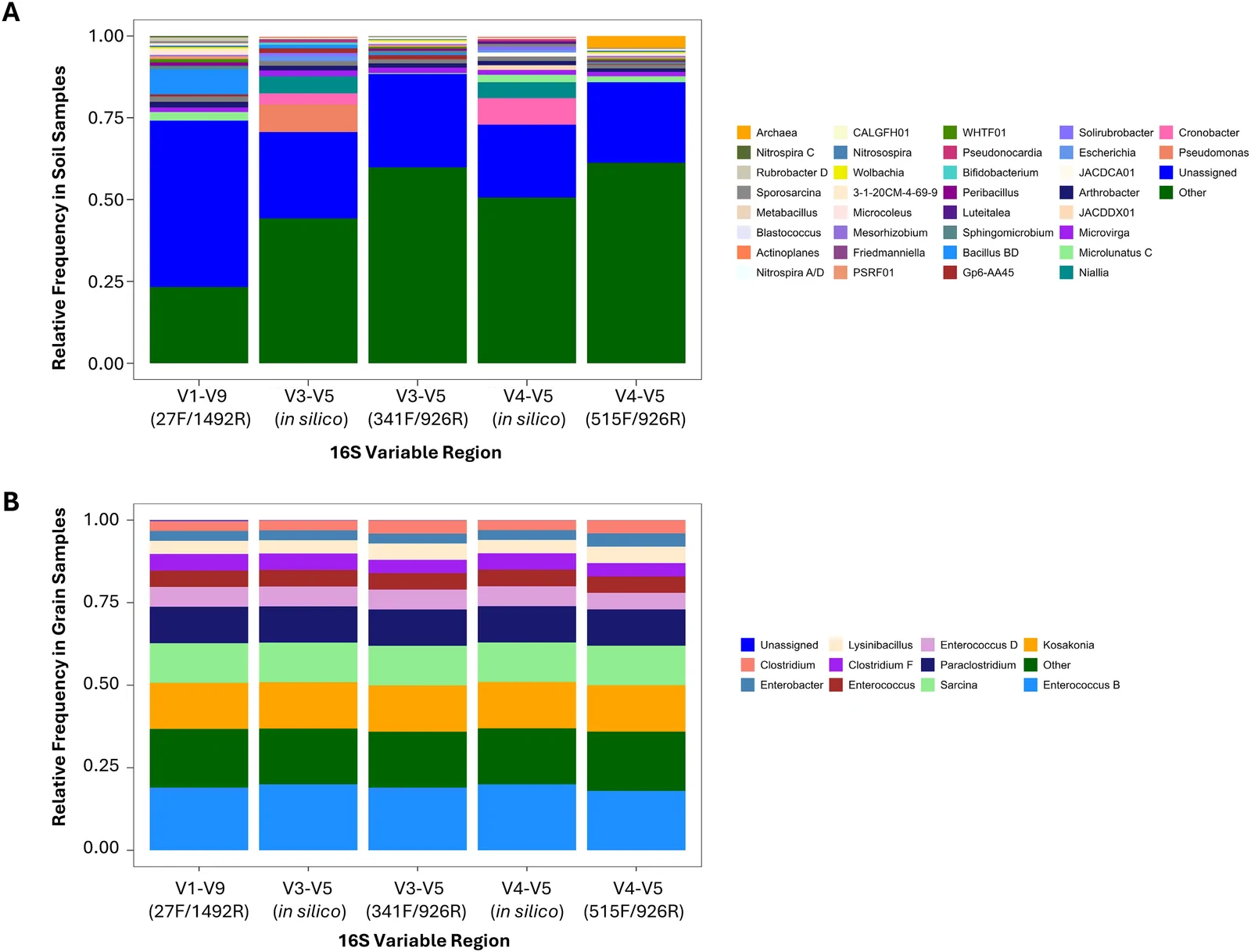
Relative frequencies of top genera assigned by VSEARCH for the top 50 ASVs for the soil (A) and top 10 ASVs for grain microbiomes (B). The three different lengths of amplicons, 27F/1492R (full-length V1-V9), 341F/926R (V3-V5) and 515F/926R (V4-V5) were investigated, and the 27F/1492R amplicon was also used to *in silico* extract the V3-V5 and V4-V5 subregions that were analyzed separately.

Overall, the analysis of ASVs supports previous studies that indicate an increased discriminatory and classification power of longer sequencing reads, which resulted in greater ASV counts despite having fewer reads [5, 6]. While the full-length (V1-V9) sequencing has benefits in terms of taxonomic classification [7, 9], it may be inappropriate to directly compare the longer amplicon to those that use different primer pairs or to *in silico* extract variable regions from longer amplicons and compare them to studies that used shorter amplicons and different primers. It is important to consider that the primers may have different binding specificities and species biases. Thus, the choice of primers that are targeting the hypervariable region(s) is important to study. For example, with many previous 16S studies examining V3-V4 [3], V4 [1, 2], and V4-V5 [4, 29] due to amplicon length limitations of short-read sequencing technologies, it remains unclear what primer biases may exist that would affect comparison of these and other studies when using the full-length amplicon. We also observed challenges in ASV assignment for the long amplicon with the soil samples, possibly due to an abundance of uncultured and undescribed microbes (Supplemental Table 3). As the 16S sequence databases and advancements in single cell biology and metagenome sequencing continue to improve, it may be valuable to revisit microbiome data sets of complex samples, particularly from environmental samples that can be prioritized based on presence of unassigned 16S sequences [31–33].

### Read-length, data processing, and community structure affect taxonomic and chimeric read classifications

PCR amplification of the full-length 16S amplicon can be complicated by several factors, including sequence similarity among taxa in complex samples, increasing amplicon length, highly variable copy numbers within taxa, the use of degenerated primers, the degree of fragmentation of template DNA, increasing PCR cycles and extension times, poor DNA polymerase quality, etc. [9, 14, 16]. When we compared the total non-chimeric sequences, 55% of the full-length amplicons from the soil samples were non-chimeric, and 86% of the V3-V5 and 92% of the V4-V5 amplicons were non-chimeric (Table 2 and Fig. 4). This trend was similar for the samples from cultures enriched from grain. Thus, increasing the amplicon length appears to increase the likelihood of chimeric reads.

**Figure 4:**
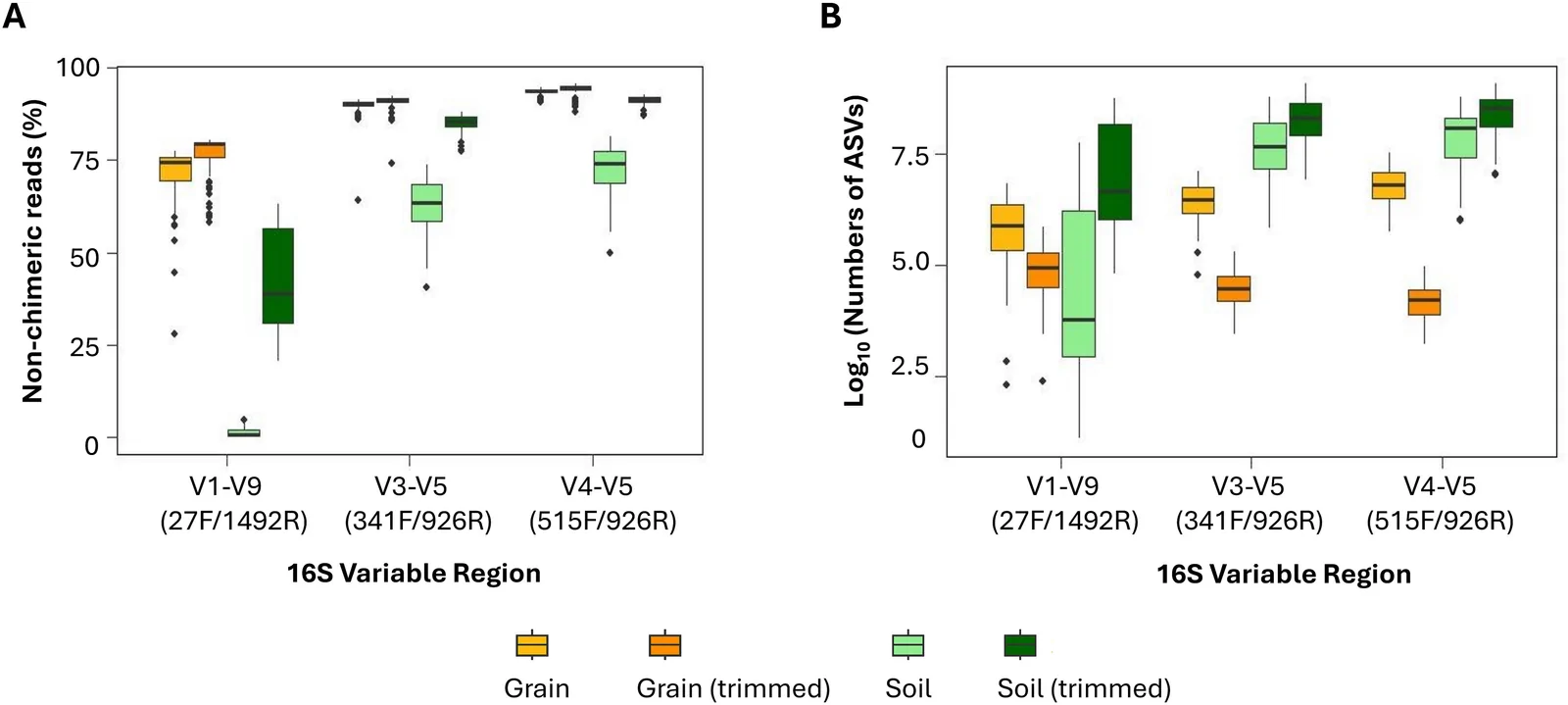
The frequency of the denoised/non-chimeric reads (A) and the total number of ASVs (B) after DADA2 analysis with skip-trim or trimmed primer pairs options. The soil and grain microbiome samples were assessed for all three amplicons.

There was a notable difference in the proportion of chimeric reads between sample types, where the soil samples had fewer non-chimeric reads than the grain enriched microbiome samples (Fig. 4). The discrepancy may reflect differences in DNA quality and sample complexity between the sample types, with the DNA from soil microorganisms being more complex (Table 2) and fragmented (Supplemental Fig. 1). Another consideration is the biases and completeness of the 16S databases, where there would be an overrepresentation of culturable samples in databases, with rare unculturable microbes having greater potential to be missing and misclassified as chimeras for the soil samples. This challenge may be overcome in time as technologies advance and datasets improve for rare unculturable microbes [31, 32].

The 16S sequencing primers often contain degenerative DNA nucleotides to expand target binding affinities for certain taxa. This may provide advantages in capturing specific taxa of interest but also leads to biases and discrepancies between studies using different primers [1–4, 7, 9, 29]. While the 16S degenerate primers are artificial sequences, they also contain meaningful variable sequences that may contribute to ASVs. To explore this further, we processed the data using DADA2 filtering and denoising with and without primer trimming for the three amplicons for both sample types (Fig. 4). In grain enriched samples, the percentage of non-chimeric sequence was only slightly reduced when retaining the primer sequences (Fig. 4A), but the number of ASVs was increased in the three amplicons, supporting the notion that the region may contain valuable information for taxon identification (Fig. 4B). When retaining the primer sequences for the soil samples, the percentage of non-chimeric sequence was reduced for the full-length amplicon; since the number of reads was significantly reduced, this resulted in a reduction in the number of ASVs. Retaining the primer sequences also reduced the number of non-chimeric reads for the other two amplicons, but a significant proportion of the reads were retained and the number of ASVs increased. In general, best practices suggest primer trimming, as they may add non-biological or synthetic sequences, which we observed to affect the number of chimeric reads for soil but not grain samples, and the number of ASVs for both sample types for all three amplicons.

On a per-dataset basis, the number of chimeric reads was greater for longer amplicons. For the soil samples, the smaller amplicons, 341F/926R (V3-V5) and 515F/926R (V4-V5), had 85% and 90% of reads pass chimeric filtering, respectively, for samples with <20 000 input sequences, which increased marginally as the number of input sequences increased (Fig. 5). In the case of the full-length 27F/1492R (V1-V9) amplicon for the soil samples, the yield of non-chimeric sequences was lower and reached ~60%, even with >40 000 input sequences (Fig. 5A). In comparison, the grain enriched samples had low numbers of ASVs (Table 2), the maximum yield of non-chimeric sequences was ~80%, 90%, and 99% with <10 000 input sequences for 27F/1492R (V1-V9), 341F/926R (V4-V5) and 515F/926R (V4-V5) amplicons, respectively. For the 27F/1492R (V1-V9) amplicon for grain enriched samples, when the input was >40 000 reads, the non-chimeric yield dropped down to ~60% (Fig. 5A). The increased chimeras could be due to the low complexity or structure of the communities in the grain samples, which is increasing the levels of chimeras in samples with higher levels of amplification, particularly when the amplicon is longer. This suggests that the number of input reads, the sample complexity/structure, DNA quality, and amplicon length may all impact the occurrence of chimeric reads. These variables are also not independent in the current study, which complicates data interpretation and inference.

**Figure 5:**
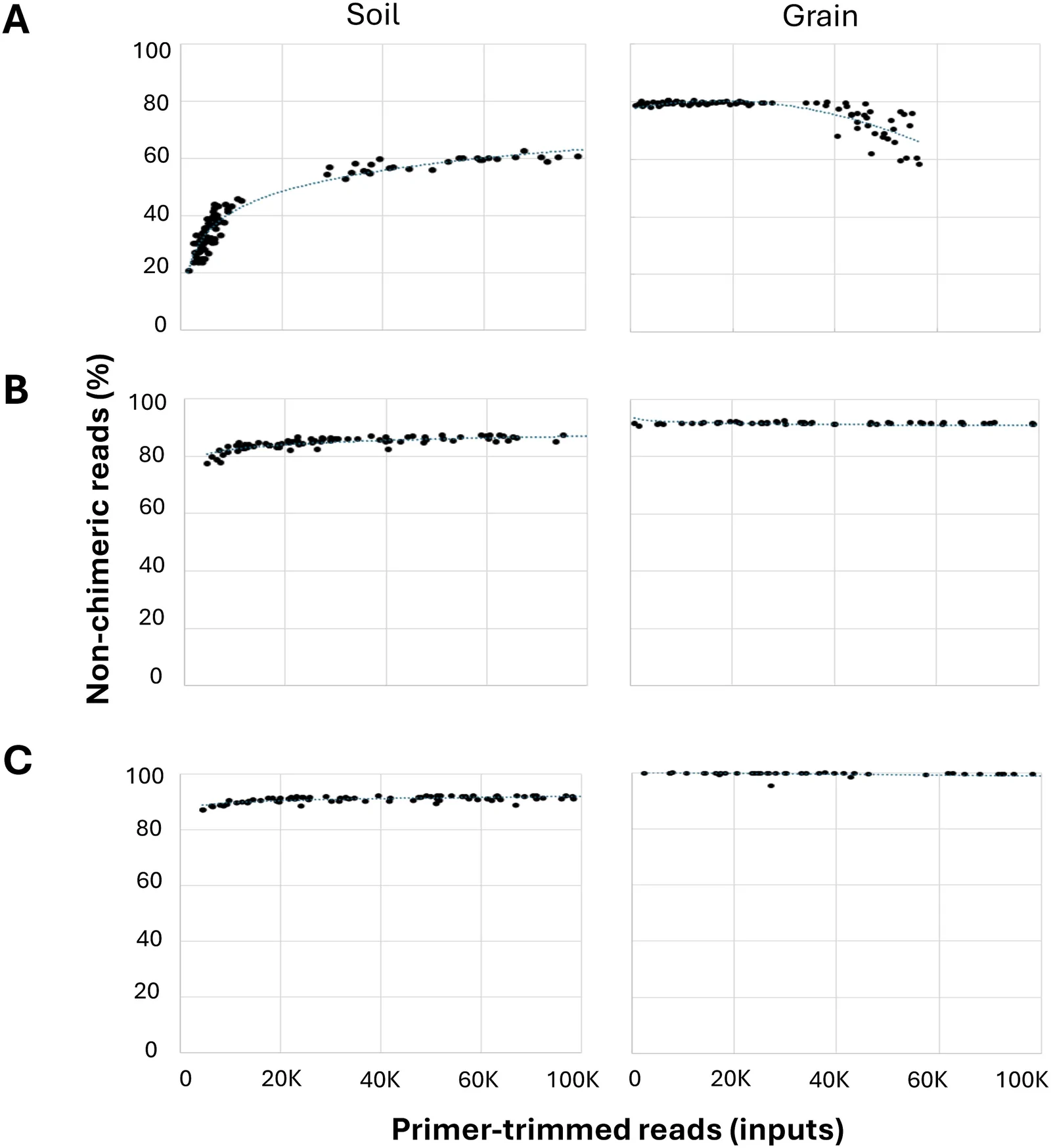
The yield plots of non-chimeric reads after the denoise and chimeric filtration by the DADA2 pipeline for the full-length (V1-V9) amplicon (A), V3-V5 amplicon (B) and V4-V5 amplicon (C). The data are presented for each of the independent datasets for the 95 grain and 96 soil microbiome datasets.

### The soil and grain-enriched microbiome samples represent unique sample types important to agriculture

Soil microbiomes often contain a diverse mixture of commensal environmental microbes, including those that may interact with plants, including field crops. For the top 50 genera in the soil samples using VSEARCH, 51% ASVs of the full-length (V1-V9) amplicons were unassigned, which was higher than the 29% of V3-V5 and 25% of V4-V5 amplicons (Fig. 3A). We had confirmed that relatively abundant genera were commonly assigned in all the three lengths of amplicons, except *Nitrospira C* (present only in the 27F/1492R amplicon), *Rubrobacter D* (not present in the 515F/926R amplicon) and archaea (assigned only using the 515F/926R amplicon). This supports the existence of bias among primer sets and amplicon lengths.

The taxonomic resolution of *Bradyrhizobium* in the soil samples is a good example of the benefits of the full-length amplicon (Fig. 6). *Bradyrhizobium* is a symbiont with various legumes, forming root-nodules for nitrogen-fixation [34, 35], and over 180 strains were detected in Australian soil metagenomes [36]. Pie charts of genus *Bradyrhizobium* in a representative soil sample were compared among the full-length, the V3-V5 *in silico* sub-region of the full-length, and the V3-V5 amplicon (Fig. 6). Based on the assignments of the full-length 16S amplicon, 11 *Bradyrhizobium* species were classified, while only 4 and 3 species were identified in the V3-V5 *in sili*co sub-region and V4-V5 amplicon, respectively. The V3-V5 *in silico* sub-region of the full-length amplicon (Fig. 6B) showed the similar pattern of *Bradyrhizobium* profile to the V3-V5 amplicon (Fig. 6C). There was a 32% unassigned portion in the full-length amplicon dataset, which was higher than the 7% of the *in silico* V3-V5 sub-region and the 3% of V3-V5 amplicon sequences. As an additional validation for the unassigned sequences, we performed a BLASTn analysis of the full-length ASVs that belonged to *Bradyrhizobium* genus to the NCBI database, and most ASVs matched to the sequences belonging to the genus *Bradyrhizobium* with high similarity. Together, the results highlight the increased taxonomic resolution achieved when capturing additional hypervariable regions within the 16S amplicon.

**Figure 6:**
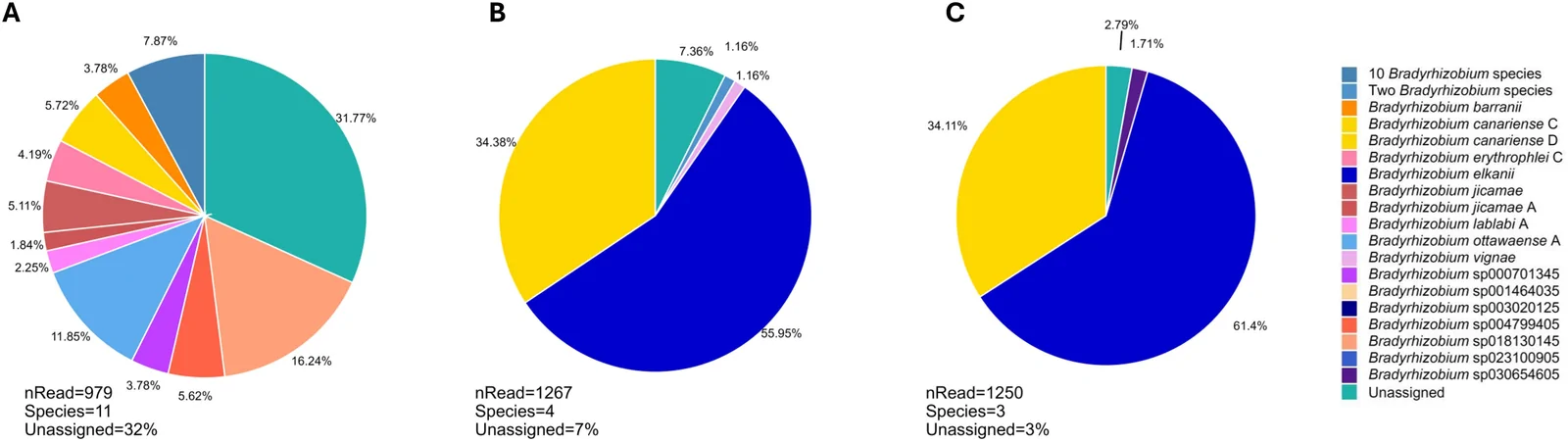
Species-level profiles of genus *Bradyrhizobium* of a soil sample. The DADA2 taxonomic analysis to the full-length 16S (V1-V9) amplicon (A), the *in silico* V3-V5 sub-region of the full-length amplicon sequence (B) and the V3-V5 region amplified by 341F/926R primers (C). The nRead indicates the numbers of amplicons in each of the pie charts.

In contrast to the soil microbiomes, there are only a few studies that have looked specifically at the microbiome of grain and the data presented here represent an underrepresented sample type [37, 38]. Seeds, including grain crops, typically contain stores of carbohydrates and proteins that act as a source of nutrients for the germinating plant when it is establishing itself after dispersal. These nutrients are also used by microbes associated with the grain. These microbes often interact with the plant, including those that may cause plant disease or promote plant health [38]. For wheat grain microbiomes, the profiles of the top 10 bacterial genera were highly consistent across the three variable regions (Fig. 3B). The genera *Enterococcus B*, *Kosakonia*, *Sarcina*, and *Paraclostridium* dominated, with 55% of the total genus representation across the three variable regions, and each of the genera constituted over 10% in relative abundance. The total number of ASVs detected increased with increasing amplicon length (Table 2). So, while the overall profiles of the top 10 genera did not appear different (Fig. 3B), many of the other genera and species were classified to a greater resolution, thus highlighting the benefits of the greater read lengths for making species or strain-level identifications.

In summary, the diverse bacterial taxa identified in both soil and grain samples in this study likely contribute important beneficial functions to the agroecosystem, supporting key biological processes that enhance soil fertility and ultimately improve agricultural productivity. Further, amplification with the longer amplicon that captures all nine hypervariable regions enhanced the taxonomic resolution when characterizing the communities.

## Conclusion

We demonstrate that the pooled sequencing of the full-length (27F/1492R, V1-V9) and the custom Kinnex combinations (341F/926R, V3-V5 and 515F/926R, V4-V5) could be successfully combined into a single library (PacBio) to enable the sequencing of all three amplicons on one flow cell (Fig. 1). The customization of the Kinnex protocol to different 16S amplicons here also provides additional precedence that customization can be applied to other amplicons and multiplexing different amplicons could prove beneficial for enhancing outputs using the accurate long-read PacBio sequencing platform. Prior to constructing the libraries, we performed an assessment of different DNA extraction and PCR conditions, which may provide guidance to other studies conducted using agricultural samples with differences in DNA quality and microbial community structure. We used these methods to explore three sample types, soil, grain, and mock microbiome samples that differed in their DNA quality and sample complexity. Several of the variables in the study were not fully independent; e.g. the soil samples were more complex in their microbial community structure and the DNA samples were more fragmented than those for the grain microbiomes, which resulted in a combination of different effects on the resulting taxonomic identifications. We observed differences in chimeric reads across amplicons, and report on the benefits of certain practices during read processing and filtering, including the trimming and removal of the primer sequences. For both soil and grain-derived samples, the longer amplicons were shown to have increased taxonomic resolution, but increased numbers of chimeric reads. Perhaps most importantly, the HiFi PacBio full-length 16S sequence data has advantages in obtaining more ASVs and more granular classifications than shorter amplicon sequences. In addition, comparison of the *in silico* subregions of the full-length amplicon to shorter amplicons across studies may be possible, but is not advised, particularly in cases where primer biases may exist that cause preferential amplification of different taxa. Further, the samples from this study represent unique microbiome samples that add to our growing knowledge on the bacteria present in different agricultural samples.

## Supplementary Material

Supplementary_material_ycag282

## Data Availability

Sequencing data has been submitted to NCBI under the BioProject Accession PRJNA1444141.

## References

[1] Bahram M, Hildebrand F, Forslund SK, et al. Structure and function of the global topsoil microbiome. *Nature* 2018;560:233–7. 10.1038/s41586-018-0386-6 30069051

[2] Caporaso JG, Lauber CL, Walters WA, et al. Global patterns of 16s rrna diversity at a depth of millions of sequences per sample. *Proc Natl Acad Sci* 2011;108:4516–22. 10.1073/pnas.1000080107 PMC3063599 20534432

[3] Hirpara KR, Hinsu AT, Kothari RK. Metagenomic evaluation of peanut rhizosphere microbiome from the farms of saurashtra regions of Gujarat, India. *Sci Rep* 2024;14:10525. 10.1038/s41598-024-61343-5 PMC11079051 38720057

[4] Labouyrie M, Ballabio C, Romero F, et al. Patterns in soil microbial diversity across europe. *Nat Commun* 2023;14:3311. 10.1038/s41467-023-37937-4 PMC10250377 37291086

[5] Aja-Macaya P, Conde-Pérez K, Trigo-Tasende N, et al. Nanopore full length 16s rrna gene sequencing increases species resolution in bacterial biomarker discovery. *Sci Rep* 2025;15:26486. 10.1038/s41598-025-10999-8 PMC12280133 40691262

[6] Zhang H, Wang X, Chen A, et al. Comparison of the full-length sequence and sub-regions of 16s rrna gene for skin microbiome profiling. *mSystems* 2024;9:e00399–24. 10.1128/msystems.00399-24 PMC11264597 38934545

[7] Johnson JS, Spakowicz DJ, Hong B-Y, et al. Evaluation of 16s rrna gene sequencing for species and strain-level microbiome analysis. *Nat Commun* 2019;10:5029. 10.1038/s41467-019-13036-1 PMC6834636 31695033

[8] Buetas E, Jordán-López M, López-Roldán A, et al. Full-length 16s rrna gene sequencing by pacbio improves taxonomic resolution in human microbiome samples. *BMC Genomics* 2024;25:310. 10.1186/s12864-024-10213-5 PMC10964587 38528457

[9] Veselovsky V, Romanov M, Zoruk P, et al. Comparative evaluation of sequencing platforms: Pacific Biosciences, oxford nanopore technologies, and illumina for 16s rrna-based soil microbiome profiling. *Front Microbiol* 2025;16:2025. 10.3389/fmicb.2025.1633360 PMC12365774 40842827

[10] O'Sullivan B, Herbst KW, Hogan AH, et al. Comparison of a long-read amplicon sequencing approach to short-read amplicons for microbiome analysis. *Microbiol Spectrum* 2026;14:e0277625–5. 10.1128/spectrum.02776-25 PMC13349200 42153723

[11] Ciuchcinski K, Bluszcz A, Dziewit L. Taxonomy, function and plasmids of microbial soil communities of polish salt graduation towers. *Sci Data* 2025;12:1702. 10.1038/s41597-025-05989-4 PMC12569027 41152332

[12] Aoki S, Weaver M, Simmonds TJ, et al. Effects of species and sex on the gut microbiome of four laboratory-reared fruit fly lines (diptera: Tephritidae) using full-length 16s rrna pacbio kinnex sequencing. *BMC Microbiol* 2025;25:455. 10.1186/s12866-025-04025-0 PMC12306104 40721996

[13] Qin H, Jiao J, Disi A, et al. Single-molecule approach to 16s rrna for vaginal microbiome signatures in response to metronidazole treatment. *Microbiol Spectrum* 2023;11:e01706–22. 10.1128/spectrum.01706-22 PMC10269914 37199621

[14] Wang GCY, Wang Y. The frequency of chimeric molecules as a consequence of pcr co-amplification of 16s rrna genes from different bacterial species. *Microbiology* 1996;142:1107–14. 10.1099/13500872-142-5-1107 8704952

[15] Child HT, Wierzbicki L, Joslin GR, et al. Comparative evaluation of soil DNA extraction kits for long read metagenomic sequencing. *Access Microbiol* 2024;6:1–19. 10.1099/acmi.0.000868.v3 PMC11432601 39346682

[16] Haas BJ, Gevers D, Earl AM, et al. Chimeric 16s rrna sequence formation and detection in sanger and 454-pyrosequenced pcr amplicons. *Genome Res* 2011:gr.112730.110;21:494–504. 10.1101/gr.112730.110 PMC3044863 21212162

[17] Baer M, Höppe L, Seel W, et al. Impact of DNA extraction, pcr amplification, sequencing, and bioinformatic analysis on food-associated mock communities using pacbio long-read amplicon sequencing. *BMC Microbiol* 2024;24:521. 10.1186/s12866-024-03677-8 PMC11622462 39643893

[18] Lee S-J, Osuna-Caballero S, Jardine L, et al. Efficient soil microbiome sampling of field crop yield trial. *protocolsio* 2026. 10.17504/protocols.io.3byl4wpd8vo5/v1

[19] Gamage NW, Bamforth J, Ashfaq T, et al. Profiling of bacillus cereus on Canadian grain. *PLoS One* 2021;16:e0259209. 10.1371/journal.pone.0259209 PMC8568128 34735500

[20] Shen W, Le S, Li Y, et al. Seqkit: a cross-platform and ultrafast toolkit for fasta/q file manipulation. *PLoS One* 2016;11:e0163962. 10.1371/journal.pone.0163962 PMC5051824 27706213

[21] Martin M . Cutadapt removes adapter sequences from high-throughput sequencing reads. *EMBnet journal* 2011;17:10–2. 10.14806/ej.17.1.200

[22] Bolyen E, Rideout JR, Dillon MR, et al. Reproducible, interactive, scalable and extensible microbiome data science using qiime 2. *Nat Biotechnol* 2019;37:852–7. 10.1038/s41587-019-0209-9 PMC7015180 31341288

[23] Callahan BJ, McMurdie PJ, Rosen MJ, et al. Dada2: high-resolution sample inference from illumina amplicon data. *Nat Methods* 2016;13:581–3. 10.1038/nmeth.3869 PMC4927377 27214047

[24] Chuvochina M, Gerken J, Frentrup M, et al. Silva in 2026: a global core biodata resource for rrna within the dsmz digital diversity. *Nucleic Acids Res* 2026;54:D334–41. 10.1093/nar/gkaf1247 PMC12807666 41251139

[25] Vinje H, Rudi K, Snipen L. Creating unity: linking 16s rrna gene sequence information to the core taxonomy of genomes. *Environ Microbiome* 2025;20:134. 10.1186/s40793-025-00789-0 PMC12570451 41153067

[26] Cole JR, Chai B, Farris RJ, et al. The ribosomal database project (rdp-ii): sequences and tools for high-throughput rrna analysis. *Nucleic Acids Res* 2005;33:D294–6. 10.1093/nar/gki038 PMC539992 15608200

[27] Velásquez-Mejía EP, de la Cuesta-Zuluaga J, Escobar JS. Impact of DNA extraction, sample dilution, and reagent contamination on 16s rrna gene sequencing of human feces. *Appl Microbiol Biotechnol* 2018;102:403–11. 10.1007/s00253-017-8583-z 29079861

[28] Curry KD, Wang Q, Nute MG, et al. Emu: species-level microbial community profiling of full-length 16s rrna oxford nanopore sequencing data. *Nat Methods* 2022;19:845–53. 10.1038/s41592-022-01520-4 PMC9939874 35773532

[29] Walters W, Hyde ER, Berg-Lyons D, et al. Improved bacterial 16s rrna gene (v4 and v4-5) and fungal internal transcribed spacer marker gene primers for microbial community surveys. *mSystems* 2015;1. 10.1128/msystems.00009-15 PMC5069754 27822518

[30] Zhao J, Rodriguez J, Martens-Habbena W. Fine-scale evaluation of two standard 16s rrna gene amplicon primer pairs for analysis of total prokaryotes and archaeal nitrifiers in differently managed soils. *Front Microbiol* 2023;14:1–12. 10.3389/fmicb.2023.1140487 PMC9995467 36910167

[31] Church DL, Cerutti L, Gürtler A, et al. Performance and application of 16s rrna gene cycle sequencing for routine identification of bacteria in the clinical microbiology laboratory. *Clin Microbiol Rev* 2020;33. 10.1128/cmr.00053-19 PMC7484979 32907806

[32] Sereika M, Mussig AJ, Jiang C, et al. Genome-resolved long-read sequencing expands known microbial diversity across terrestrial habitats. *Nat Microbiol* 2025;10:2018–30. 10.1038/s41564-025-02062-z PMC12313526 40707831

[33] Rieder J, Kapopoulou A, C B, et al. Metagenomics and metabarcoding experimental choices and their impact on microbial community characterization in freshwater recirculating aquaculture systems. *Environ Microbiome* 2023;18:8. 10.1186/s40793-023-00459-z PMC9930364 36788626

[34] Stacey G, Sanjuan J, Luka S, et al. Signal exchange in the bradyrhizobium-soybean symbiosis. *Soil Biol Biochem* 1995;27:473–83. 10.1016/0038-0717(95)98622-U

[35] Zhong C, Hu G, Hu C, et al. Comparative genomics analysis reveals genetic characteristics and nitrogen fixation profile of bradyrhizobium. *iScience* 2024;27:108948. 10.1016/j.isci.2024.108948 PMC10845061 38322985

[36] Bueno, de Mesquita CP, Olm MR, Bissett A, et al. High strain-level diversity of bradyrhizobium across australian soils. *ISME J* 2025;19:1–12. 10.1093/ismejo/wraf222 PMC12596133 41060304

[37] Michl K, Kanasugi M, Förster A, et al. The microbiome of a perennial cereal differs from annual winter wheat only in the root endosphere. *ISME Commun* 2025;5:1–12. 10.1093/ismeco/ycae165 PMC11812607 39936170

[38] Sun Z, Adeleke BS, Shi Y, et al. The seed microbiomes of staple food crops. *Microb Biotechnol* 2023;16:2236–49. 10.1111/1751-7915.14352 PMC10686132 37815330

