# Supplementary material for: Comparison of long and short 16S rRNA gene amplicon sequencing for agricultural soil and grain microbiomes using custom Kinnex libraries
Source: ISME Commun. 2026 Oct 6;6(1):ycag282. doi: 10.1093/ismeco/ycag282 (PMC13712307; doi:10.1093/ismeco/ycag282)
Supplement: Supplementary file 1 [file supplementary_material_ycag282.zip › Supplemental Figures.pdf]

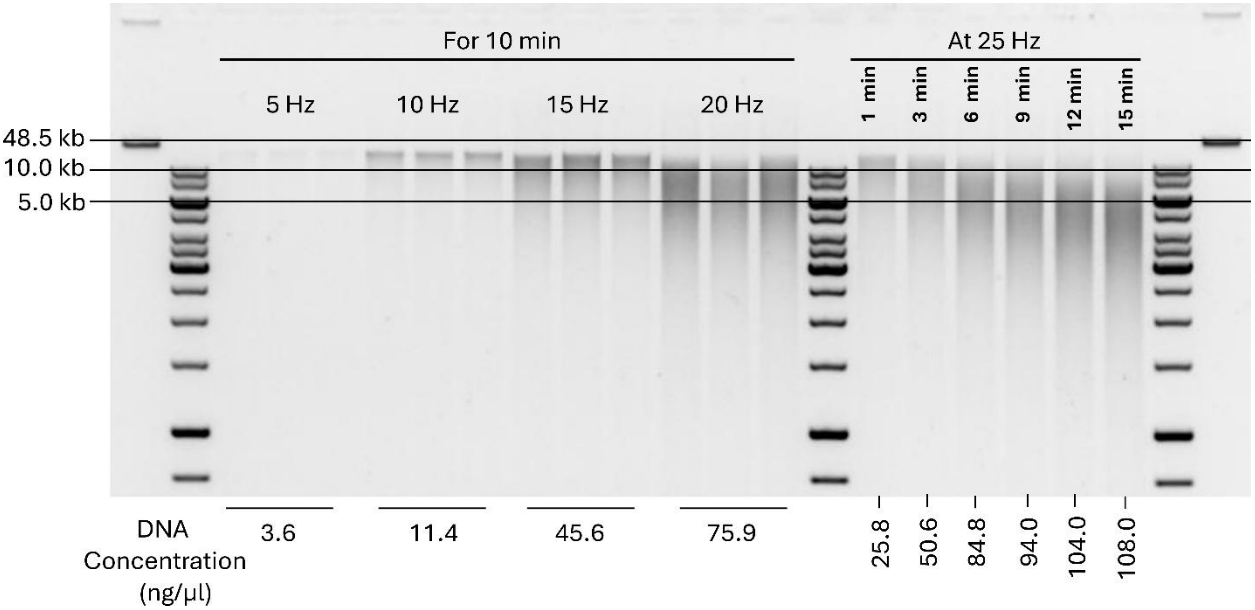

Supplemental Figure 1 The DNA fragmentations in the variations of the shaking speeds and durations in the Mixermill (Retch) using DNeasy PowerSoil Pro kit (Qiagen). The DNA yield at the bottom is the concentration as measured by the Qubit HS kit (ThermoFisher Sci.) from a 100 µL elution.

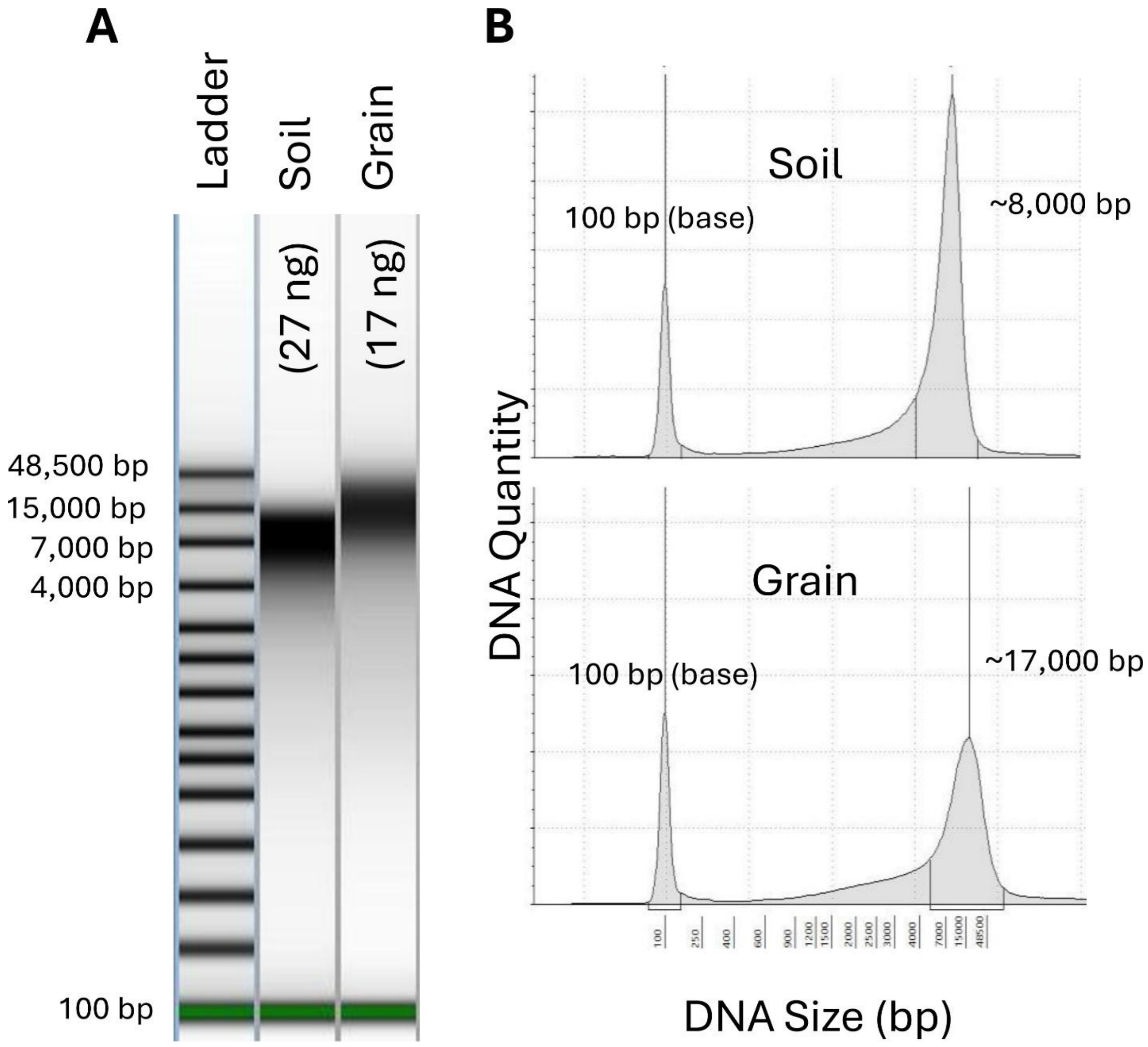

Supplement Figure 2 Size distribution of soil and grain microbiome DNA used in this study. Synthetic gel image (A) and size distributions (B) were generated using TapeStation Genomic DNA ScreenTape Analysis (Agilent).

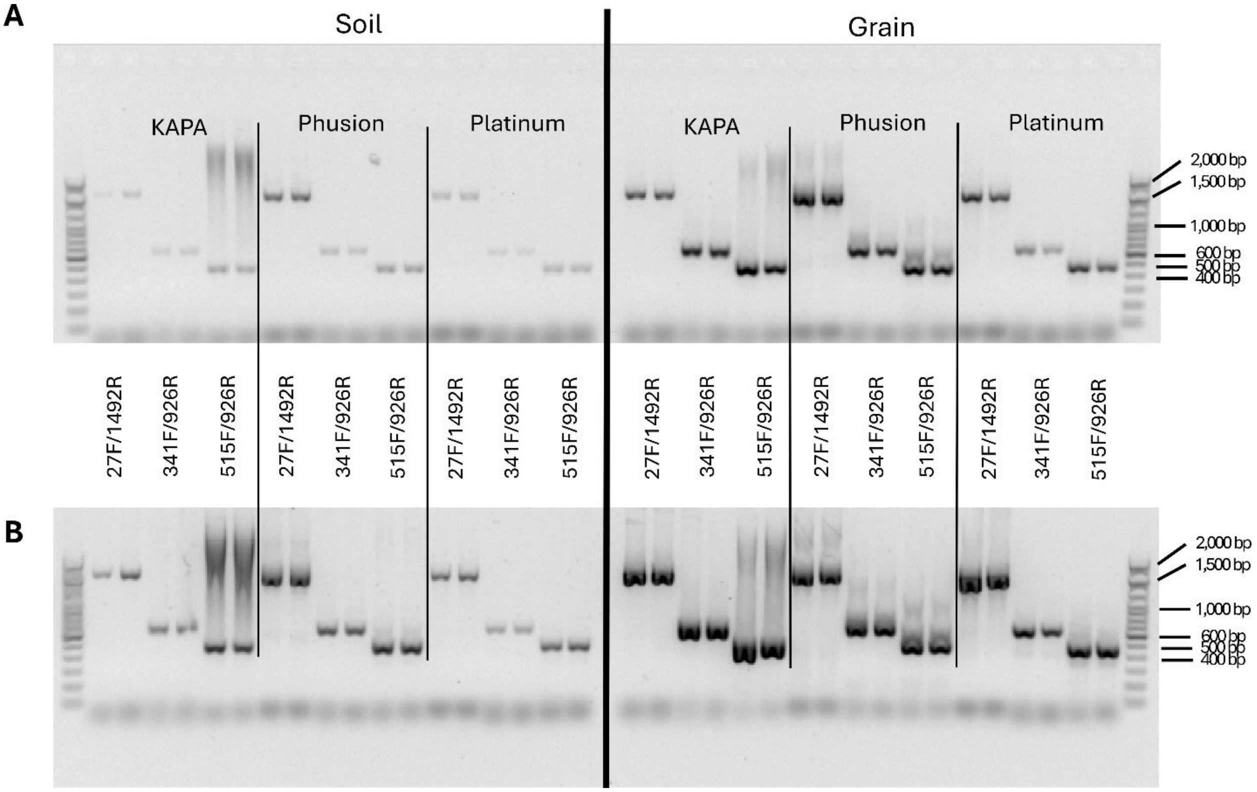

Supplement Figure 3 The PCR products amplified by the Kinnex barcoded primer pairs, 27F/1492R (V1-V9), 341F/926R (V3-V4) and 515F/926R (V4-V5), from the soil DNA (left) and grain microbial community DNA (right), respectively. The 20 cycles (A) and 23 cycles (B) at 57 ˚C annealing temperature recommend by the full-length 16S Kinnex protocol (PacBio). KAPA: KAPA HiFi HotStart ReadyMix (Roche), Phusion: PhusionHotStart II HiFi PCR MasterMix (ThermoFisher Sci.) and Platinum: Platinum HotStart PCR MasterMix (ThermoFisher Sci.).
